# Assessing the perceived impact of ESOR training programs on radiologists’ professional development

**DOI:** 10.1186/s13244-024-01891-6

**Published:** 2025-02-17

**Authors:** Jules Grégory, Mathias Kofoed-Ottesen, Brigitte Lindlbauer, Christian Loewe, Valérie Vilgrain, European Society of Radiology (ESR)

**Affiliations:** 1 Imaging department, Beaujon Hospital, FHU MOSAIC & APHP. Nord, Paris Cité Université, Clichy, France; 2 European School of Radiology Office, Wien, Austria; 3 Department of Biomedical Imaging and Image-Guided Therapy, Medical University Vienna, Wien, Austria; 4 Am Gestade 1, Vienna, Austria

**Keywords:** European Society of Radiology, Education (medical), Professional development, Training support, Specialization

## Abstract

**Objectives:**

This study evaluates the perceived impact of European School of Radiology (ESOR) training programs on radiologists’ professional development.

**Methods:**

A cross-sectional survey targeted alumni who participated in ESOR fellowships from 2011 to 2023. The survey included questions on demographics, professional background, ESOR program details, and career impact. Data were collected via a web-based questionnaire and analyzed using descriptive statistics and thematic analysis.

**Results:**

A total of 916 alumni were invited to the survey, with a response rate of 21% (190 participants). The median age was 31 years (range 29–33), and 54% were female. Most worked in public healthcare (62%) and were involved in academic activities (24%). Fellowship types included the visiting scholarship program (44%), Bracco fellowship (32%), and exchange program for fellowships (25%). The majority (59%) reported the fellowship helped them reach their current position, and 35% noted it upgraded their CV. Significant application of learned skills was reported by 69%. Ongoing cooperation with former tutors was maintained by 54%. Financial support was crucial, with 41% stating they could not have completed the training without it, 33% considering it very important, and 13% important. Participants rated the impact on clinical skills with a median score of 9/10. Other areas of impact included research skills (median 7/10), subspecialization (median 9/10), exposure to diverse practices (median 9/10), networking (median 10/10), and personal and professional growth (median 10/10).

**Conclusion:**

ESOR training programs significantly enhance radiologists’ professional development through comprehensive support, high-quality training, and substantial financial aid, ensuring participants are well-equipped for career advancement.

**Critical relevance statement:**

This study evaluates the perceived impact of ESOR training programs on radiologists’ professional development, highlighting significant enhancements in clinical skills, career advancement, and the critical role of financial support in facilitating access to high-quality education.

**Key Points:**

- The ESOR offers various programs addressing both foundational and advanced training in radiology.
- Fifty-nine percent of participants reported that ESOR fellowships helped them achieve their current positions.
- Participants experienced a median improvement score of 9 out of 10 in clinical skills.
- Fifty-four percent of participants maintained ongoing cooperation with former tutors post-fellowship.
- ESOR financial support was perceived as crucial by many participants, ensuring access to high-quality education.

**Graphical Abstract:**

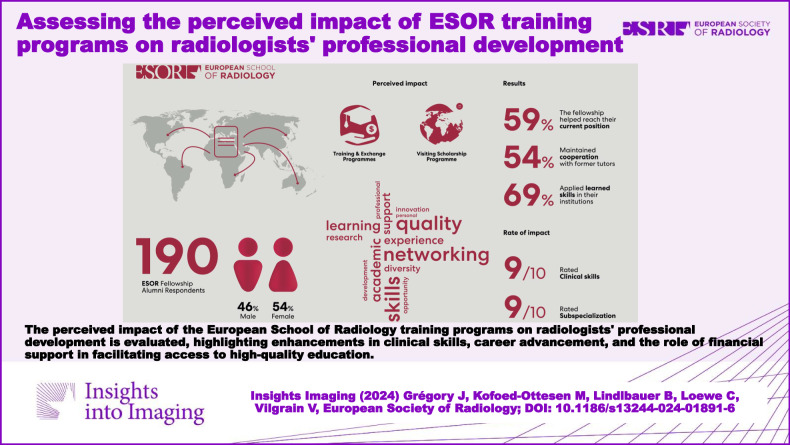

## Introduction

The European School of Radiology (ESOR) plays a pivotal role in the ongoing education and training of radiologists across Europe [1, 2]. Established to enhance the professional competencies and knowledge of radiologists at various stages of their careers, ESOR offers a multitude of meticulously structured programs. These programs are designed to provide foundational and advanced training, facilitating both personal and professional growth in the field of radiology.

ESOR’s training and exchange programs serve a diverse group of radiologists, from residents to recently certified professionals. These programs provide both foundational and advanced training in various radiology subfields, including modality-specific and subspecialty-focused training, interactive workshops, and practical skills courses. The exchange programs offer invaluable exposure to different clinical practices across Europe, fostering a comprehensive understanding of radiological procedures and innovations. Investing in mobility promotes essential collaboration among centers, preventing isolation, is vital for multicentric evidence-based medicine, unifies techniques and terminology across countries, and is crucial for developing shared guidelines [3]. The Erasmus program exemplifies European mobility for trainees, facilitating cross-border knowledge exchange and enhancing collaborative efforts to advance standards and practices [4].

For residents and recently certified radiologists, ESOR provides several fellowship opportunities that are crucial for further specialization and career advancement. The visiting scholarship program in Europe enables radiology residents to spend three months in leading European radiology departments, gaining insights and learning from top experts. The exchange programs for fellowships in partnership with European radiological subspecialties (three-month training) and the Bracco fellowships (two-month training) offer specialized training in diagnostic or interventional imaging, allowing participants to gain expertise in advanced imaging techniques and procedures. More recently, other programs have been developed as well: The Nicholas Gourtsoyiannis Teaching Fellowship, named in honor of a prominent radiologist, focuses on teaching skills and curriculum development, preparing radiologists for academic and educational roles and the multidisciplinary visiting fellowship program, the aim of which is to strengthen the multidisciplinary relationship between radiologists and clinicians by offering a tandem in-depth immersion in a radiology department and a clinical department with exposure to complex clinical cases [5]. Additionally, one-year or two-year fellowships overseas in Europe have been developed to provide longer-term, in-depth training. These fellowships are more limited in number but offer extensive training and research opportunities, allowing radiologists to fully immerse themselves in their chosen subspecialty.

The application process for ESOR training programs involves submitting a standardized curriculum vitae (CV) and a letter of motivation. The evaluation of applicants is conducted by two jury members using a standardized evaluation grid. This grid assesses various criteria, including the quality of the letter of motivation, the applicant’s CV, scientific publications, letters of recommendation, and previous involvement in ESOR or European Congress of Radiology (ECR) activities.

Given the rigorous application and evaluation process, the ESOR programs are highly regarded for their ability to significantly enhance the clinical and research skills of radiologists [6]. These programs also foster international collaboration and networking, contributing substantially to the advancement of radiology as a field.

This study aims to evaluate the perceived impact of ESOR training programs on the professional development of radiologists. By focusing on the subjective experiences and career advancements of participants, this research seeks to provide valuable insights into the efficacy and benefits of ESOR’s educational initiatives.

## Methods

### Study design and participants

A cross-sectional survey was designed, targeting alumni who participated in any of ESOR’s fellowship programs between 2011 and 2023, encompassing a total of 916 radiologists. Ethical approval was not required for this survey as it involved professional feedback without collecting sensitive personal data. All participants were informed about the purpose of the study and the anonymity of their responses.

### Survey instrument

The survey, developed under the leadership of Professor Valérie Vilgrain and the ESOR board, was designed to capture the experiences of participants and assess the impact of ESOR training programs on their professional trajectories.

The questionnaire included structured and open-ended questions capturing demographic information (age, gender, and country of origin), professional background (current employment setting and academic activities), details of ESOR program participation (program attended, year, and type), and the perceived impact on career advancement.

It also assessed skill acquisition and application within participants’ home institutions, the development of professional collaborations, and the role of financial support in facilitating program participation. Additional areas of inquiry focused on enhancements in clinical and research skills, growth in subspecialty knowledge, experiences in diverse medical environments, opportunities for networking and personal development, and scholarly contributions influenced by the training received.

### Data collection

The survey was sent via email to the 916 ESOR alumni, containing a link to the web-based questionnaire hosted on a commercial platform (SurveyMonkey©). The survey was open for a period of 8 weeks from September 2023. Reminder emails were sent to potential respondents to enhance the response rate.

### Statistical analysis

Responses were collected and analyzed using R software (version 4.2). Quantitative data were analyzed to derive frequencies, percentages, and median values, while qualitative responses were thematically analyzed to identify prevalent themes related to the training’s impact and feedback.

### Supplemental material

The original survey questionnaire is provided as Electronic Supplemental Material.

## Results

A total of 190 participants (21% response rate) completed the survey, with a median completion time of 8 min and 41 s (IQR: 5:28–13:25).

### Participant characteristics

**Fig. 1:**
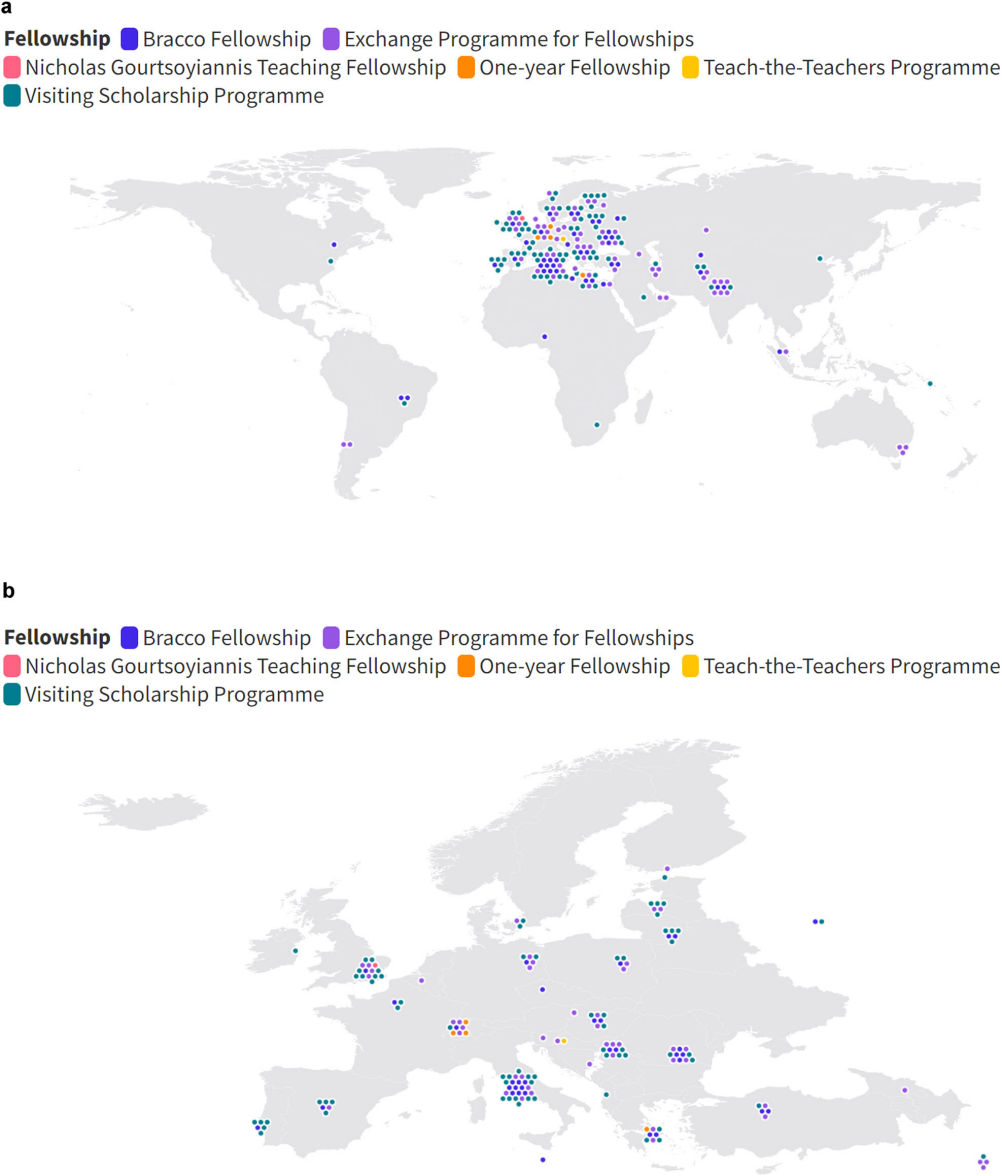
Geographical distribution of the ESOR fellowship participants. Each dot represents a participant, with different colors indicating different programs. **a** Global distribution of ESOR fellowship participants. **b** European distribution of ESOR fellowship participants

Out of the 190 participants who responded to the survey, 54% were female. The median (Q1–Q3) age of the participants was 31 years (29–33). The majority of participants (62%) worked in public healthcare settings, 13% in private practice, 24% in mixed settings, and 1% were fully engaged in research activities. Additionally, 24% of participants were involved in academic activities at the time of the survey. The geographic distribution of participants spanned multiple countries, with the highest representations from Italy (17%), Romania (6%), and the United Kingdom (6%) (Fig. 1 and Table 1).

**Table 1:** Participants demographics and geographic distribution of ESOR alumni participants

Participant characteristics	Number (%) of participants (total = 190)
Gender	
• Female	102 (54)
• Male	88 (46)
Participant age (median [Q1–Q3])	31 (29–33)
Current professional practice	
• Public	118 (62)
• Private	25 (13)
• Mixed	46 (24)
• Full research	1 (5)
Current academic activity	45 (24)
Country of residence*	
Italy	33 (27)
Romania	12 (30)
United Kingdom	11 (35)
Serbia	9 (39)
Greece	8 (17)
Switzerland	8 (50)
Spain	7 (11)
Turkey	7 (16)
Hungary	6 (30)
India	6 (14)
Lithuania	6 (43)
Germany	5 (19)
Portugal	5 (26)
Afghanistan	4 (100)
Australia	4 (80)
Denmark	4 (100)
Iran	4 (33)
Latvia	4 (50)
Brazil	3 (12)
Poland	3 (9)
Belgium	2 (10)
Chile	2 (33)
Croatia	2 (11)
Czech Republic	2 (25)
Egypt	2 (29)
France	2 (33)
Nigeria	2 (29)
Russia	2 (12)
Slovakia	2 (40)
Slovenia	2 (12)
The Netherlands	2 (33)
United Arab Emirates	1 (100)
Albania	1 (25)
Bosnia and Herzegovina	1 (100)
Canada	1 (100)
China	1 (5)
Finland	1 (100)
Georgia	1 (14)
Ireland	1 (33)
Kazakhstan	1 (50)
Luxembourg	1 (100)
Malaysia	1 (25)
Malta	1 (50)
Mexico	1 (20)
North Macedonia	1 (100)
Singapore	1 (50)
Thailand	1 (33)
United States	1 (100)
Uzbekistan	1 (50)

* Percentages represent the proportion of alumni from each country relative to the total number of ESOR fellows awarded, not the percentage of survey respondents

### ESOR fellowship participation

Participants had attended all types of ESOR fellowship programs over the years, with the number of attendees increasing over time. The highest number of participants was in 2018 (15%), followed by 2022 (11%), and 2019 (11%). The majority of participants (85%) attended only one program, while 12% attended two programs, and 3% attended three programs. The types of fellowships attended included the visiting scholarship program (44%), Bracco fellowship (32%), and exchange program for fellowships (25%). Additionally, a small number of participants attended other specialized programs such as the one-year fellowship, teach-the-teachers program, and Nicholas Gourtsoyiannis teaching fellowship. Participants became aware of the ESOR training programs through various channels, including word-of-mouth (17%), ECR events (33%), ESOR visiting professorship programs (1%), mailing lists (20%), the ESOR website (66%), internet searches (15%), and social media (6%) (Table 2).

**Table 2:** ESOR fellowship participation details

ESOR fellowship attended	Number (%) of participants (total = 190)
Year of participation in ESOR fellowship*	
2011	10 (17)
2012	8 (12)
2013	13 (15)
2014	11 (13)
2015	9 (15)
2016	8 (11)
2017	15 (20)
2018	28 (24)
2019	21 (19)
2020	5 (9)
2021	2 (15)
2022	20 (38)
2023	16 (14)
Number of programs attended	
1	162 (85)
2	23 (12)
3	5 (3)
Fellowship participation	
• Visiting scholarship program	83 (44)
• Bracco fellowship	60 (32)
• Exchange program for fellowships	47 (25)
Additional program(s)	
• One-year fellowship	6 (3)
• Teach-the-teachers program	2 (1)
• Nicholas Gourtsoyiannis teaching fellowship	1 (.5)
Awareness of the ESOR training program**	
• Word-of-mouth advertising	33 (17)
• At ECR	63 (33)
• During an ESOR visiting professorship program	2 (1)
• Through a mailing	38 (20)
• ESOR website	125 (66)
• Google/internet	29 (15)
• Social media	12 (6)

* Percentages represent the proportion of alumni for each year relative to the total number of ESOR fellows awarded, not the percentage of survey respondents for that year

** Categories are not exclusive

### Impact of ESOR programs

The impact of the ESOR fellowships on participants’ career development was significant, with 59% reporting that the fellowship helped them reach their current position and 35% noting it upgraded their CV. In terms of applying learned skills, 69% reported significant application in their home institutions, and 54% maintained ongoing cooperation with their former tutors post fellowship.

The ESOR programs had a notable impact on participants’ clinical skills, with a median improvement score of 9 out of 10. Other areas of impact included research skills (median score 7), subspecialization advancement (median score 9), exposure to diverse medical practices (median score 9), networking opportunities (median score 10), and personal and professional growth (median score 10). Additionally, 75 participants reported publication output related to their fellowship, and 74 submitted conference abstracts (Table 3).

**Table 3:** Professional and academic impact of ESOR fellowships

Feedback on the ESOR fellowship	*N* = 190
Impact of fellowship on career development	
• Yes, for sure, it helped me reach my current position	112 (59)
• Yes, it definitely upgraded my CV	66 (35)
• Uncertain	4 (2)
• No, not at all	3 (2)
• NA	5 (3)
Application of learned skills in the home institution	
• A lot	132 (69)
• Part of	46 (24)
• Not much	7 (4)
• NA	5 (3)
Continued cooperation post-fellowship	
• Yes, I am still in touch with my former tutor	103 (54)
• Yes, I was in touch with my former tutor, but I am not anymore now	34 (18)
• No	45 (25)
• NA	5 (3)
Significance of financial grant received	
• I could not have done the training without it	77 (41)
• Very important	63 (33)
• Important	24 (13)
• Somewhat important	14 (7)
• Not that important as I had also other financial support	7 (4)
• NA	5 (3)
Impact of the ESOR fellowship (from 10 [most] to 1 [least]) (median [Q1–Q3])	
• Improvement in clinical skills	9 (8–10)
• Enhancement of research skills	7 (5–10)
• Subspecialization advancement	9 (8–10)
• Exposure to diverse medical practices	9 (8–10)
• Networking and contact expansion opportunities	10 (8–10)
• Personal and professional growth experience	10 (9–10)
• Acquaintance with new cultures and environments	10 (9–10)
Publication output related to fellowship	75 (39)
Conference abstract submissions related to fellowship	74 (39)
Fulfillment of ESOR program expectations	
• Yes, it exceeded my expectations	94 (49)
• Yes, for sure	74 (39)
• More or less	12 (6)
• Not really	2 (1)
• Not at all	3 (1.6)
• NA	5 (3)
Contribution of ESOR programs to intellectual and social enrichment	184 (99)

### Subgroup analysis

The subgroup analysis of the ESOR fellowship programs shows that the visiting scholarship program had the highest positive impact, with 62% of participants acknowledging that the fellowship helped them reach their current position, and 71% reporting significant application of their acquired skills in their home institutions. For the exchange program for fellowships, 57% indicated career advancement and 65% applied the skills learned extensively. Among participants of the Bracco fellowship, 59% reported that the fellowship helped them reach their current position, while 70% could apply a lot of what they learned in their home institutions.

Continued cooperation with former tutors was most notable among Bracco fellowship participants (58%), compared to 53% for the visiting scholarship program and 51% for the exchange program. The median improvement in clinical skills was highest in the visiting scholarship and Bracco programs (both at 9), with similar scores observed for research skills, subspecialization, and personal growth across all programs.

The analysis of expectations and intellectual enrichment showed that 62% of participants in the visiting scholarship program felt the program met their expectations, with 34% stating it exceeded their expectations, and 2% saying it more or less met their expectations. None reported unmet expectations. In the exchange program for fellowships, 57% reported the program met their expectations, 38% said it exceeded expectations, and 4% felt it more or less met their expectations. For the Bracco fellowship, 59% indicated the program met their expectations, 35% felt it exceeded their expectations, 3% noted it more or less met their expectations, and 2% reported unmet expectations (Table 4).

**Table 4:** Subgroup analysis of professional and academic impact by fellowship type for ESOR programs

Feedback on the ESOR fellowship	Visiting scholarship program (total: 75)	Exchange program for fellowships (total: 52)	Bracco fellowship (total: 63)
Impact on career development			
• Yes, for sure, it helped me reach my current position	47 (62)	30 (57)	37 (59)
• Yes, it definitely upgraded my CV	26 (34)	20 (38)	22 (35)
• Uncertain	2 (2)	2 (4)	2 (3)
• No, not at all	0 (0)	0 (0)	1 (2)
• NA	0 (0)	0 (0)	1 (2)
Application of learned skills			
• A lot	53 (71)	34 (65)	44 (70)
• Part of	15 (20)	14 (26)	14 (22)
• Not much	5 (7)	4 (7)	4 (6)
• NA	2 (2)	1 (2)	1 (2)
Continued cooperation post-fellowship			
• Yes, I am still in touch with my former tutor	40 (53)	27 (51)	37 (58)
• Yes, I was in touch, but I am not anymore	15 (20)	11 (21)	15 (24)
• No	18 (24)	13 (25)	9 (15)
• NA	2 (3)	1 (2)	2 (3)
Importance of financial grant received			
• Indispensable	32 (43)	20 (39)	26 (42)
• Very important	23 (30)	18 (35)	20 (32)
• Important	11 (15)	7 (13)	9 (14)
• Somewhat important	5 (7)	4 (8)	4 (7)
• Not important	2 (3)	2 (4)	3 (4)
• NA	2 (2)	1 (1)	1 (1)
Improvement in clinical and research skills (median [Q1–Q3])			
• Clinical skills	9 [8–10]	8 [7–10]	9 [8–10]
• Research skills	7 [5–10]	6 [4–9]	7 [5–10]
• Subspecialisation	9 [8–10]	8 [7–10]	9 [8–10]
• Exposure to different medical practices	9 [8–10]	8 [7–10]	9 [8–10]
• Networking opportunities	10 [9–10]	9 [8–10]	10 [8–10]
• Personal and professional growth	10 [9–10]	9 [8–10]	10 [9–10]
• Knowledge of new cultures	10 [9–10]	9 [8–10]	10 [9–10]
Expectations and intellectual enrichment			
• Met expectations	47 (62)	30 (57)	37 (59)
• Exceeded expectations	26 (34)	20 (38)	22 (35)
• More or less met expectations	2 (2)	2 (4)	2 (3)
• Not met expectations	0 (0)	0 (0)	1 (2)
• NA	0 (0)	0 (0)	1 (2)

### Financial support

Financial support played a crucial role in enabling participants to attend the ESOR programs. Overall, 41% of participants stated that they could not have completed the training without financial grants, 33% considered the financial support very important, and 13% deemed it important (Table 3). In a detailed analysis by program, financial grants were considered indispensable by 43% of visiting scholarship recipients, 42% of Bracco fellows, and 39% of Exchange Program participants (Table 4).

### Thematic analysis

The thematic analysis of participant feedback identified four key domains for improvement: host center obligations (mentioned 12 times), personalized training plans (mentioned 8 times), extended duration (mentioned 6 times), and enhanced feedback mechanisms (mentioned 5 times). Participants suggested stricter obligations for host centers to ensure consistent and high-quality training, with clearly defined roles and expectations for both hosts and participants. They also highlighted the need for more personalized training plans that align with individual career goals and specific radiological interests. Additionally, extending the duration of certain programs was proposed to allow for a deeper understanding and mastery of complex procedures. Finally, participants recommended improved feedback mechanisms, emphasizing the need for more structured sessions to facilitate better learning outcomes.

**Fig. 2:**
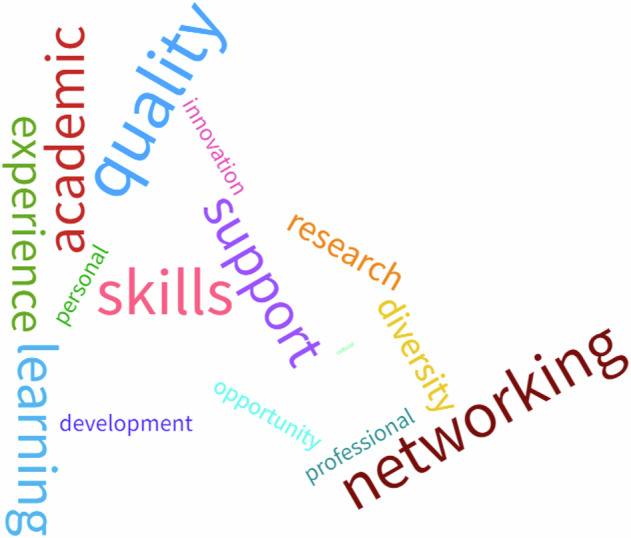
Word cloud depicting key positive themes from ESOR training program feedback. This figure illustrates a word cloud generated from the qualitative analysis of feedback provided by participants of the ESOR training programs. The word cloud highlights the key positive themes identified from the feedback, with the size of each word corresponding to the frequency of its occurrence in the participants’ responses

Positive feedback centered on three main domains: professional development (mentioned 15 times), academic career advancement (mentioned 10 times), and cultural exchange (mentioned 7 times). Many participants reported significant improvements in clinical skills, research capabilities, and overall professional growth, attributing these advancements to the ESOR training programs. The training was also noted to positively influence academic careers, leading to new teaching opportunities, research projects, and publications. Additionally, the cultural exchange aspect of the programs was highly valued, with participants appreciating the exposure to diverse medical practices and environments (Fig. 2).

## Discussion

The findings of this study highlight the significant positive impact of ESOR training programs on the professional development of radiologists. These programs cater to radiologists at different stages of their careers, with the visiting scholarship program providing early career exposure during residency, while the exchange program for fellowships and Bracco fellowship offers advanced training opportunities post-residency. This structured approach ensures that radiologists receive continuous support throughout their professional journey, enhancing both foundational and specialized skills. The consistent performance across the visiting scholarship program, Bracco fellowship, and exchange program for fellowships suggests a well-structured and effective support system within the ESOR organization. The importance of structured training programs for professional development has been emphasized in emerging medicine and gastroenterology, underscoring the universal value of such initiatives [7].

The rigorous selection process for candidates is a critical factor contributing to the success of ESOR training programs. By maintaining high selection standards, ESOR ensures that only the most motivated and capable radiologists are chosen. This selective process not only enhances the overall quality of the program but also ensures that participants are likely to fully engage with and benefit from the training provided. The selection process evaluates candidates’ academic and professional backgrounds, ensuring that those selected are well-prepared to make the most of the training opportunities.

The quality of training centers is pivotal to the effectiveness of ESOR programs. These centers, among the best in Europe, are equipped with state-of-the-art facilities and staffed by leading experts in various radiology subspecialties. Some are certified under the European Training Assessment Program (ETAP) 2.0, ensuring high standards through structured assessment [8]. The training centers undergo continuous evaluation to ensure they meet the highest standards of education and clinical practice. Additionally, feedback is solicited from both tutors and tutees, ensuring a comprehensive evaluation of the training experience. Based on the results of this study, to further enhance training consistency and quality, a charter for host centers has been established, clearly defining roles and expectations for both hosts and participants. This commitment to quality ensures that participants receive top-notch training and are exposed to the latest advancements in radiology.

Another essential aspect is the interdisciplinary nature of training. In each department, the rich learning environment is fostered by multidisciplinary exchanges. Each tutor is dedicated to a single tutee, ensuring focused guidance while the department’s diverse specialties broaden the participant’s knowledge and skills. This aligns with the recent findings by the European Society of Radiology, which emphasize the importance of structured, high-quality training programs for radiology residents in Europe, including the development and implementation of entrustable professional activities [9].

The career trajectories of former participants underscore the long-term benefits of ESOR training programs. A significant proportion of alumni have pursued academic careers, reflecting one of ESOR’s primary objectives to enhance academic excellence in radiology. These collaborations offer targeted training opportunities in specific areas of radiology, combining the expertise and resources of multiple organizations to provide comprehensive educational experiences. The program’s emphasis on long-term collaboration rather than a one-time experience is evident, as many participants continue to engage in professional relationships and collaborative projects initiated during their fellowships. This aligns with the success of mentoring programs like the National Research Mentoring Network (NRMN), which emphasizes sustained mentorship and professional growth, particularly for underrepresented groups [10].

The financial support provided to participants is another key factor in the success of ESOR programs. This funding alleviates the financial burden associated with advanced training and international mobility, making it possible for a wider range of participants to benefit from these opportunities. The financial backing is crucial as it ensures that talented radiologists, regardless of their economic background, can access high-quality training and further their careers. Robust financial support systems are essential for fostering diversity and inclusion in training programs, as seen in various initiatives aimed at addressing workforce diversity [10].

Robust mentorship, as highlighted by the National Research Mentoring Network (NRMN), has been linked to enhanced mentee productivity, self-efficacy, and career satisfaction, which are critical to the success of researchers in training [11, 12]. Effective mentorship programs must create a supportive environment with mentors and peers committed to advancing the careers of investigators from traditionally underrepresented groups, addressing the disparities highlighted by studies on NIH funding barriers [13].

The COVID-19 pandemic temporarily halted international exchanges, providing an opportunity to rethink and improve these programs. Now, the number of exchanges has returned to pre-pandemic levels, ensuring continued collaboration and knowledge sharing across borders [14, 15]. Despite the significant successes and positive impacts of ESOR training programs, it is essential for ESOR not to rest on its laurels but to continually strive for innovation and improvement, using an educational structured framework such as the Kern model of curricular development [16]. By involving systematic steps such as identifying needs, setting objectives, developing educational strategies, implementation, and evaluation, ESOR could establish specialized working groups tailored to different target audiences. These groups would be responsible for continuously assessing the needs of their respective audiences, ensuring that the training programs remain relevant.

This study has several limitations that should be acknowledged. First, the cross-sectional design relies on self-reported data from participants, which may introduce response bias and limit the accuracy of the reported outcomes. Participants’ recollections and perceptions of the impact of ESOR training programs might be influenced by personal experiences and subjectivity. In addition, for participants who completed the program more recently, the full impact of the fellowship may not yet be apparent, particularly in terms of career development. Second, the survey sample, while diverse, may not fully represent all alumni of the ESOR programs, potentially limiting the generalizability of the findings. The response rate was 21%, which is relatively low and may impact the representativeness of the results due to non-response bias, where the views of non-respondents might differ from those of respondents, thereby skewing the results. Additionally, while the survey captured a wide range of perceived impacts, it did not directly quantify specific career advancements or objectively measure skill improvements, such as the Clinically Oriented Reasoning Evaluation (CORE) [17]. Furthermore, although the programs investigated share a common educational goal, their differences in duration and structure may lead to variability in the outcomes, and this heterogeneity should be considered when interpreting the results.

In conclusion, the success of ESOR training programs can be attributed to the comprehensive support they offer throughout different career stages, a rigorous candidate selection process, high-quality training centers, and substantial financial support. These factors collectively ensure that participants are well-supported, highly motivated, and receive excellent training. The notable career achievements of program alumni, particularly in academic roles, further validate the effectiveness of these programs in meeting their objectives and advancing the field of radiology.

## Supplementary information

ELECTRONIC SUPPLEMENTARY MATERIAL

## Data Availability

The datasets generated and/or analyzed during the current study are not publicly available due to privacy constraints but are available from the corresponding author upon reasonable request.

## Abbreviations

CV: Curriculum vitae
ECR: European Congress of Radiology
ESOR: European School of Radiology
