# Supplementary material for: Assessing the perceived impact of ESOR training programs on radiologists’ professional development
Source: Insights Imaging. 2025 Feb 17;16:34. doi: 10.1186/s13244-024-01891-6 (PMC11833024; doi:10.1186/s13244-024-01891-6)
Supplement: Supplementary file 1 — ELECTRONIC SUPPLEMENTARY MATERIAL [file 13244_2024_1891_MOESM1_ESM.pdf]

Assessing the perceived impact of ESOR training programs on radiologists' professional development

ELECTRONIC SUPPLEMENTARY MATERIAL

Insights Imaging (2024) Grégory J, Kofoed-Ottesen M, Lindlbauer B, Loewe C, Vilgrain V.

ESOR Survey

---

1. Name of your home institution and department
[Free text field]

2. City
[Free text field]

3. Country
[Free text field]

4. Title of your current position
[Free text field]

5. Which of the categories below best describe your current position?
You can choose more than one answer.

- ☐ Private Practice
- ☐ Public Practice
- ☐ Mixed Practice
- ☐ A non-academic position
- ☐ An academic position (please describe your position type, e.g., assistant professor, full professor, etc.)
  [Free text field]

6. Date of birth
Pick from calendar.

---

7. When did you go on which exchange programme(s)?
If you went on multiple programmes, please mark multiple answers.

- in 2007: [Dropdown menu]
- in 2008: [Dropdown menu]
- in 2009: [Dropdown menu]
- in 2010: [Dropdown menu]

- in 2011: [Dropdown menu]
- in 2012: [Dropdown menu]
- in 2013: [Dropdown menu]
- in 2014: [Dropdown menu]
- in 2015: [Dropdown menu]
- in 2016: [Dropdown menu]
- in 2017: [Dropdown menu]
- in 2018: [Dropdown menu]
- in 2019: [Dropdown menu]
- in 2020: [Dropdown menu]
- in 2021: [Dropdown menu]
- in 2022: [Dropdown menu]
- in 2023: [Dropdown menu]

---

8. How did you come to know about the ESOR Training Programme?
Please tick off any applicable answer.

- ☐ Word of mouth advertising
- ☐ At ECR
- ☐ During an ESOR Visiting Professorship Programme
- ☐ Through a mailing
- ☐ ESOR website
- ☐ Google/Internet
- ☐ Social Media (Facebook, LinkedIn, Instagram)
- ☐ Other (please specify):
  [Free text field]

---

9. Did the scholar-/fellowship have any impact on your career development?

- ( ) Yes, for sure, it helped me reach my current position
- ( ) Yes, it definitely upgraded my CV
- ( ) I don't know
- ( ) I am not sure
- ( ) No, not at all

10. How much of what you have learned during your ESOR scholar-/fellowship have you been able to apply back in your home institution?

- ( ) A lot
- ( ) Part of it
- ( ) Not much
- ( ) Nothing at all

11. Has the ESOR scholar-/fellowship led to a continued cooperation between your home institution and you/your host institution?

- ( ) No
- ( ) Yes, I am still in touch with my former tutor
- ( ) Yes, I was in touch, but I am not anymore now (please specify how long you remained in contact with your tutor):
  [Free text field]

---

12. How important was the financial grant which you received in connection with your scholar-/fellowship?

- ( ) I could not have done the training without it
- ( ) Very important
- ( ) Important

- ( ) Somewhat important
- ( ) Not that important as I had also other financial support
- ( ) Not important at all, I would have done the training also without financial contribution from ESOR

---

13. What is the most important advantage/benefit you had from your participation in the ESOR Training Programme(s)?
Please rate the options from most (10) to least (1) important.

- I improved my clinical skills
- I improved my research skills
- Subspecialisation
- I discovered other practices
- It was a great opportunity to network and to expand my personal radiology contacts
- It was a great personal experience that allowed me to broaden my mind
- I got to know a new city/country and culture
- Other (please specify):
  [Free text field]

---

14. Have you published any articles/manuscripts during or shortly after and related to your scholarship/fellowship period?

- ( ) No
- ( ) Yes (please specify which article(s) and in which journal(s)):
  [Free text field]

15. Have you submitted any abstracts to any congress following your scholar/fellowship?
If yes, please specify which abstract(s) and to which congress(es):

- ( ) No

- ( ) Yes (please specify):
  [Free text field]

16. Did the ESOR Training Programme(s) you completed meet your expectations?

- ( ) Yes, it/they exceeded my expectations
- ( ) Yes, for sure
- ( ) More or less
- ( ) Not really
- ( ) Not at all
  Any comment: [Free text field]

17. Do you feel the ESOR Training Programmes are supporting intellectual and social enrichment?

- ( ) Yes
- ( ) No
  Any comment: [Free text field]

---

18. Do you have any comments/suggestions in general on how to improve the ESOR Training Programmes?
[Free text field]
